# Cell wall glucans of fungi. A review

**DOI:** 10.1016/j.tcsw.2019.100022

**Published:** 2019-03-21

**Authors:** José Ruiz-Herrera, Lucila Ortiz-Castellanos

**Affiliations:** Departamento de Ingeniería Genética, Unidad Irapuato, Centro de Investigación y de Estudios Avanzados del Instituto Politécnico Nacional, Km. 9.6, Libramiento Norte, Carretera Irapuato-León, 36821 Irapuato, Gto. Mexico

**Keywords:** Alpha glucans, Beta glucans, Fungi, Cell wall, Phylogenetic analyses

## Abstract

Glucans are the most abundant polysaccharides in the cell walls of fungi, and their structures are highly variable. Accordingly, their glucose moieties may be joined through either or both alpha (α) or beta (β) linkages, they are either lineal or branched, and amorphous or microfibrillar. Alpha 1,3 glucans *sensu strictu* (pseudonigerans) are the most abundant alpha glucans present in the cell walls of fungi, being restricted to dikarya. They exist in the form of structural microfibrils that provide resistance to the cell wall. The structure of beta glucans is more complex. They are linear or branched, and contain mostly β 1,3 and β 1,6 linkages, existing in the form of microfibrils. Together with chitin they constitute the most important structural components of fungal cell walls. They are the most abundant components of the cell walls in members of all fungal phyla, with the exception of Microsporidia, where they are absent.

Taking into consideration the importance of glucans in the structure and physiology of the fungi, in the present review we describe the following aspects of these polysaccharides: *i*) types and distribution of fungal glucans, *ii*) their structure, *iii*) their roles, *iv)* the mechanism of synthesis of the most important ones, and *v*) the phylogentic relationships of the enzymes involved in their synthesis.

## Highlights

• Glucans are the most abundant compounds in the fungal cell walls.
• The most common type of glucose bonding is 1 → 3, both alpha and beta.
• Microfibrillar glucans with chitin provide rigidity to the fungal wall.
• Fungal beta glucans act as PAMPS during infection of animals and plants.

## Graphical abstract

**Figure:**
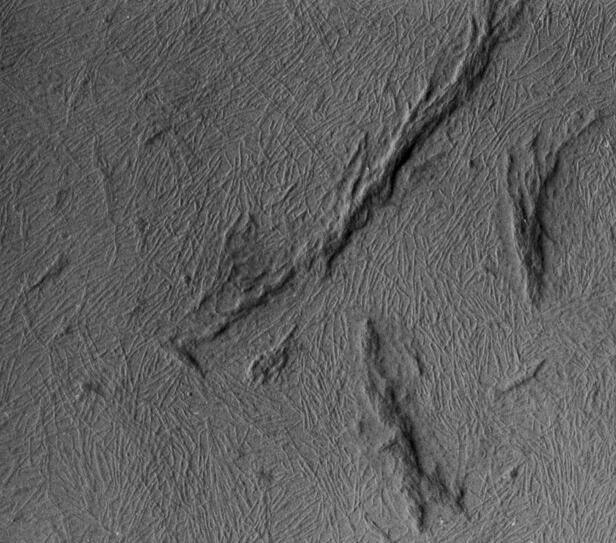

## 1 Introduction

### 1.1 The relative importance of polysaccharides in nature

All living organisms are constituted by four kinds of macromolecules: nucleic acids made of nitrogen bases, and pentoses; proteins made of amino acids, lipids made mostly of glycerol and fatty acids, and polysaccharides made of simple sugars (carbohydrates). In the whole, it has been calculated that the annual production of carbohydrates widely exceeds the rest by a large proportion. Thus, the production of carbohydrates is close to 75% of the total annual biomass production, followed by lignin (20%). In contrast, the sum of the annual production of nucleic acids, proteins and lipids, makes up only 5%. Of the total production of carbohydrates, only 3.5% (6 billion tons) are used by humans, mostly as food (62%), followed by use for paper and construction (33%), and clothing, chemicals, etc. (5%) (Kamm et al., 2008).

These data clearly point out the quantitative importance of carbohydrates for the maintenance of life. The last examples stress the qualitative importance of carbohydrates for the conservation of all forms of life on Earth as indicated above.

### 1.2 Glucans, their abundance, and their different types

Interestingly, the most abundant polysaccharides in nature are made of glucose, that is, they are glucans. It has been widely repeated that cellulose is the most abundant organic compound on Earth. Glucans are named according to their type of linkage and the carbons involved in their joining, or receive specific names. In general, glucans are divided into two large classes: alpha bound glucans and beta bound glucans, whose characteristics and functions are rather different. In general, some alpha glucans are amorphous and soluble in hot water, and play a role as energy reserve material. Examples of them are glycogen in fungi and animals, and starch in algae and plants, but those present in the fungal walls are water-insoluble and microfibrilar. Microfibrils are formed by the association of several glucan chains through hydrogen bonding. The role of microfibrillar polysaccharide is mostly structural. Most beta glucans are insoluble in water and almost all solvents, they are mostly crystalline, and can form microfibrils made as indicated above for alpha glucans. Examples of beta glucans are cellulose in plants, and different β1,3 glucans in fungi.

## 2 Characteristics of fungal glucans

### 2.1 General characteristics of alpha glucans in fungi

It is known that there exist several types of alpha glucans in fungi. The most common of these polysaccharides are: glycogen, that is made of glycosyl units joined by α 1,4 and α 1,6 bonds, and glucans in which the glycosyl units are bound solely through α 1,3 bonds, or α 1,3 and α 1,4 bonds (Bacon et al., 1968; Gold et al., 1973, Hochstenbach et al., 1998, Grün et al., 2005). As mentioned above, glycogen is a material of energy reserve that is accumulated in the form of grains in the cytoplasm. Polysaccharides of the other classes are components of the cell wall, where they are structural elements (Hochstenbach et al., 1998, Grün et al., 2005).

### 2.2 General characteristics of beta glucans in fungi

In contrast to alpha glucans, the classes of fungal beta glucans are more abundant, and their structures are more variable.

Most fungal beta glucans are made of β 1,3 joined glucose units with variable amounts of β 1,6 joined branches. However, at least one example of a β 1,3 glucan containing β 1,2 besides β 1,6 joined branches was described.

The general structures of the different types of fungal beta glucans were described by Ruiz-Herrera (1992). Seven different classes were apparent: *i*) β 1,3 unbranched glucans, *ii*) β 1,3 glucans, with occasional β 1,6 branches made of a single glucose unit; *iii*) glucans containing β 1,3 and β 1, 4 joined glucose moities; *iv*) glucans containing β 1,3, β 1,4, and β 1,6 joined moieties; *v*) β 1,3, phosphorylated glucans; *vi*) β 1,3 glucans with significant β 1,6 branching; and *vii*) glucans containing mostly β 1,6 bound glucose units. Examples of each class were described, and it was noticed that most fungal species contain laminarin. Laminarin is a mostly linear beta glucans made of glucosyl units joined by β 1,3 bonds. Some lichen fungi contain linear polysaccharides made of β 1,3 and β 1,4 bound glucosyl units, and pustulan, a partially water-soluble polysaccharide made of glucosyl units bound by β 1,6 linkages described to be present in the lichenized Ascomycotina Umbilicariaceae (Narui et al., 1999). Pustulan from *Lasallia pustulata* was described to be partially acetylated (Pereyra et al., 2003).

Most authors agree that cellulose is absent in the members of the kingdom Fungi, but a report from Schweige-Hufnagel et al. (2000) described the presence of a polysaccharide made by β 1,4 joined glucose units, *i.e.,* cellulose, in the fungus *Microdichium nivale.* This report has not been confirmed nor denied further on, but recently, Kang *et al.* (2018) identified a glucan containing β1,4 bonds in *Aspergillus fumigatus* by solid-state NMR. It is important to mention that an instructive review of the methods used for extraction, purification, and the chemical methods used to characterize alpha glucans from edible mushrooms was published by Ruthes et al., (2015). Their application to glucans from other fungi would be extremely valuable.

## 3 Fungal wall alpha glucans

### 3.1 Distribution and types of alpha glucans

Fungal species contain a number of different types of alpha glucans. Most of them are made of glucose moieties bound by α 1,3 linkages that incidentally have received a name different of the straightforward designation of alpha 1,3 glucans. For example, the name of pseudonigeran is repeatedly found in the literature (see below). Other common alpha glucans are nigeran made of alternating α 1,3 and α 1,4 bound glucose moieties (see below). Even alpha 1,3 glucans with a few α 1,4 branches have been described*.* One example is a glucan present in the fruiting bodies of *Lentinus edodes* (Shida et al., 1978).

Probably the most thorough taxonomic analysis of alpha glucans in fungi based on their chemical structure can be found in the review made by Synytsya and Novák (2013). These authors divide α-d-glucans according to their structure in the following classes: *i*) Linear α-d-glucans, divided in those having exclusively α 1,3, α 1,4 or α 1,6 bonds, and those that contain several of these types of bonds. In the first group of α 1,3 bound glucans they cite pseudonigeran, probably the most common alpha glucan, Amylose was the cited example of an α1-4 glucan. The authors also cited glucans containing solely α 1,6 bonds present in the basidiocarps of *Sarcodon aspratus* and *Termitomyces eurhizus*. Among the class of glucans containing mixed types of alpha bonds, they cite nigeran that consists of alternating maltotriose units of α 1,4 linked glucose moieties, bound by α 1–3 bonds; isolichenan, present in several lichens, with a similar structure to nigeran, but with different proportions of α 1,4 and α 1,3 bonds. The cited example of α 1,6 glucans was pullulan, initially described in *Aureobasidiun pullulans*. ii) Branched α-d-glucans. The most common polysaccharide of this class is glycogen, made, as already described, by glucose linked through α 1,4 bonds with α 1,6 bound branches. As mentioned above, this is a common reserve material in fungi. Other similar polysaccharides contain glucose bound by α 1,4 bonds, and α 1,6 bound branches made of a single glucose molecule, for example a polysaccharide from *Tricholoma matsutake*. A more detailed description of some of these polysaccharides is made below.

As described above, alpha 1,3 glucans *sensu strictu* (pseudonigerans) are probably the most abundant alpha glucans present in the cell walls of fungi, but interestingly, as described below, these polysaccharides are specific of Dikarya, and accordingly they are absent in the species belonging to the lower fungal phyla.

Possibly, the best studied example of an α 1,3 glucan is the one present in the wall of *Schizosaccharomyces pombe*, where it accounts to a smaller, but significant amount of the whole glucans of the cell wall compared to β 1,3 glucans: 28% and 42–50% respectively. This polysaccharide is present in the cell wall of this fungus accumulated close to the cell membrane and at the outer parts of the wall (Sugawara et al., 2003). Regarding its structure, it was described that the α 1,3 glucan in *S. pombe* exists in the form of a polymer made of two interconnected glucose linear chains made of about 120 glucose units each, linked by α 1,3 bonds with a few α 1,4 bonds at the reducing end (Grün et al., 2005).

As described above, pullulan is a polysaccharide made of α 1,4 and α 1,6 bound glucose units. Most studies on this polysaccharide have been made in the black yeast *A. pullulans*, although it is not specific of this species, Accordingly its presence in other fungi has been reported. Among these we may cite: Tremella mesenterica, *Cytaria hariotti, Cryphonectria parasitica, Teloschistes flavicans*, and *Rhodotorula bacarum*, (reviewed by Singh and Saini, 2011).

The structure of pullulan from *A. pullulans* was analyzed by Catley et al., 1986, Dragica Jakovljevi et al., 2001. Fine structural analyses of the fungal polysaccharide pullulan elaborated by *Aureobasidium pullulans*, CH-1 strain showed that it is made of maltotriose units bound by terminal α 1,6 bond glucose, with a small number of randomly located maltotetraose units. It is important to notice that pullulan has potential industrial applications, and that accordingly the analysis of the factors involved in its production by *A. pullulans* has been the subject of a number of studies (*e.g.* Cheng et al., 2011).

Another alpha glucan present in the cell wall of fungi is Nigeran, a polysaccharide present in the surface of several fungi, *e g.* different *Aspergillus a*nd *Penicillium* species (*e.g.* Gupta and Mukergi, 1982), and made, as described above, of glucose units linked through α 1,3 and α 1,4 bonds, and deeply inserted within the cell wall (Tung and Nordin, 1967). An analysis of the tetrasaccharide obtained by its hydrolysis with mycodextranase, confirmed that it was made by alternating glucose units bound by α 1,3 and α 1,4 bonds (Tung and Nordin, 1968). Interestingly, wall-associated nigeran was found to be crystalline and accordingly very resistant to enzymatic attack (Bobbitt et al., 1977).

It was described that synthesis of nigeran was increased by depletion of the carbon and nitrogen sources in the growing media of the different fungal species, and that metal ions, and especially copper, increase its accumulation (Gupta and Mukergi, 1982).

### 3.2 Structure of alpha glucans

There exist a limited number of studies dealing with the structure of alpha glucans. Of them, most studies deal with the most abundant form of them (pseudonigerans), and involve the analysis of the polysaccharide present in Ascomycota mostly. Accordingly, the structure of α 1,3 glucan from *S. pombe* as described by Grün et al (2005) appeared in the form of two linear chains made of *ca*. 120 α 1,3-linked glucose units, with some α 1, 4 bonds at the reducing end. Electron microscopic studies have revealed that it appears in the form of microfibrils 10–14 nm thick, *e.g.* the polysaccharides from *Paracoccidioides brasiliensis*, *Aspergillus niger*, *Polyporus betulinus* and *Histoplasma farciminosum* (Carbonell et al., 1970, San Blas and Carbonell, 1974). In the Basidiomycota species *Armillaria mellea*, an alkali-soluble α 1,3 glucan appeared also in a microfibrillar form (Sanchez-Hernandez et al., 1993).

Regarding the structure of pullulan, whose composition was described above, it is made of maltotriosyl units, and occasional maltotetraosyl units bound by α 1,6 linkages. It was suggested that the polysaccharide exists in the form of linear molecules with a molecular weight of about 40 kDa (Catley et al., 1986).

### 3.3 The roles of alpha glucans

α 1,3 Glucan appears to be non-essential in *Aspergillus nidulans*, *Schizophyllun commune*, and *Aspergillus fumigatus* (Sietsma and Wessels, 1988, Zonneveld, 1972, Zonneveld, 1973, Henry et al., 2012). In the latter species, mutation in the three genes encoding the synthesizing enzymes could be compensated by an increase in the synthesis of β1,3 glucan and chitin (Henry et al., 2012). But on the other hand, in *Paracoccidioides brasiliensis, Histoplama capsulatum, Blastomyces dermatitidis*, and *Cryptococcus neoformans*, α 1,3 glucans are more abundant in the pathogenic yeast form, and appear to be required for their normal morphology and virulence. In *P. brasiliensis* it was observed that loss of α 1,3 glucan by sub-cultivation in culture media led to the reduction in virulence, that was regained when the amounts of the polysaccharide increased by inoculation in animal hosts (San-Blas et al., 1977). It was also reported that avirulent mutant strains of *H. capsulatum* lacking the polysaccharide were avirulent (Klimpel and Goldman, 1988), and that inhibition of α 1,3 glucan synthesis by RNA interference led to virulence reduction (Rappleye et al. 2004). Similarly, in the case of *B. dermatitidis* it was described that mutants that were almost avirulent had rather nil levels of α 1,3 glucan (Hogan and Klein, 1994). Another example of the important role of α 1,3 glucans is the observation that loss of the polysaccharide in *C*. *neoformans* affected the cell structure and virulence of the fungus (Reese et al., 2007). In addition, it was reported that inhibition of the enzyme responsible for α 1,3 glucan synthesis in this fungus, inhibited the formation of the capsule (Reese and Doering, 2003). It is well known that *C. neoformans* capsule is an essential virulence factor, and the authors suggested that this observation pointed out a possible alternative for the treatment of cryptococcosis.

In conection to these results it is interesting to recall the role of beta glucans in the recognition of invading fungi by their hosts, either animals or plants. This recognition leads to the deployment of their defense mechanisms (see below). This reaction has led to denominate these compounds as pathogen-associated molecular patterns (PAMPs). According to this, it was shown that the role of α 1,3 glucan in the virulence of *Histoplasma capsulatum* is to block the recognition of β 1,3 glucans by the host specific receptor (dectin; Rappleye et al. 2004). Another example of this role by α 1,3 glucans was reported by Fujikawa et al. (2012) on phytopathogenic fungi. These authors described that α 1,3 glucan covers the surface of the cell wall of the rice pathogen *Magnaporthe oryzae*, facilitating the infection of the host, while mutants deficient in the α 1,3 glucan synthase *Mg AGS1*, were unable to penetrate into the rice plant. Moreover, the authors observed that transgenic rice plants that secreted a bacterial α 1,3 glucanase were resistance not only to *M. oryzae*, but also to *Cochliobolus miyabeanus* and *Rhizoctonia solani*. All these results suggested that the α 1,3 glucan layer protects the invading hyphae from host recognition, and their digestion by the degradative enzymes produced by the plant.

Not only virulence, but also development may be affected by alterations in the levels of alpha glucans in the cell. Accordingly, it was described that formation of cleistothecia in *A. nidulans* was dependent on the levels of α 1,3 glucan. If the amounts of the polysaccharide were low, no cleistothecia were formed (Zonneveld, 1972). Another example of wall alterations by the levels of α 1,3 glucan was the observation that overexpresion of the synthesizing enzyme increased cellular adhesion and wall defects of *A. nidulans* (He et al., 2018).

Regarding pullulan, a relationship between production of the polysaccharide and morphology of *A. nidulans* was described. Thus, it was observed that the synthesis of pullulan varied during the life cycle of the fungus, being higher in the spore and the yeast form of the fungus, whereas the mycelium produced much lower amounts of the polysaccharide (Catley, 1980).

### 3.4 Synthesis of alpha glucans.

Probably the best studied mechanism of synthesis of an alpha glucan corresponds to α 1,3 glucan. The first demonstration of the genetic control of the synthesis of this polymer in *S*. *pombe* revealed that a gene denominated as *AGS1* encoded the synthesizing enzyme (Hochstenbach et al., 1998). A temperature sensitive (*ags1*-*1^ts^*) mutant showed that its morphology was affected by temperature: at low temperature (19 °C), the cells had a rather normal morphology, but at 34 °C they were spherical, and if incubated at 37 °C, the cells were sensitive to osmotic shock and lysed. As expected, at this temperature their levels of α 1,3 glucan were very low (Hochstenbach et al., 1998). These results indicated the important role of α 1,3 glucan in cell morphology and wall resistance. The authors also cloned the gene encoding Ags1, a protein with a MW of 272 kDa. This protein contains three major hydrophobic regions with a single transmembrane region that separates anextracellular domain at the amino terminus, and an intracellular region located towards the carboxy terminus. These results were further on confirmed by Katayama et al (1999) who used a similar approach to the one employed by Hochstenbach et al (1998), in order to identify the gene encoding α 1,3 glucan synthase. These authors described the existence of not one, but a family made of five genes encoding α 1,3 glucan synthases. Of this, one was further analyzed receiving the denomination of *MOK1* (the reason for this peculiar denomination, and not the logical one given before of *AGS,* Alpha Glucan Synthase is unknown). These authors also analyzed the possible regulation of the cloned gene, suggesting that a protein kinase C (Pkc2) was involved in the localization of the enzyme. Later on Calonge et al. (2000) reported that the synthezing process was more complicated, involving a role of Rho2 for activation of Pkc2.

Interestingly, it was further reported that not one, but three members of the 1,3 glucan synthase family were involved in the synthesis of the polymer in the spores of *S*. *pombe* (García, et al., 2006).

The multiplicity of α 1,3 glucan synthases is not specific of *S*. *pombe,* there are species having more than two, and up to seven genes as occurs in *A. niger*. An interesting observation is that there are many species of Basidiomycota that have no α 1,3 glucans in their cell walls, and therefore have no α 1,3 glucan synthases. The rest of the analyzed species have only one or at most two genes with the exception of *R. solani* that has four genes encoding α 1,3 glucan synthases.

Regarding the possible different roles of the enzymes in the species having more than one α 1,3 glucan synthase, in *A. nidulans* where the presence of five genes encoding α 1,3 glucan synthases was reported, one of them (*AGS*A) was induced by stress to the cell wall, suggesting that it might be involved in wall structure conservation (Damveld et al., 2005). Similar results were described for *A. fumigatus*, where three *AGS* genes were identified (Beauvais et al., 2013). Interestingly, growth and spore germination were not affected in a triple mutant, but its virulence was decreased, and displayed severe alterations in the cell wall surface.

In the same line, two *AGS*A plus one *AGS*B genes, were discovered to encode the most important enzymes involved in the synthesis of the polysaccharide in *A. niger*. Nevertheless, one *AGS*A gene was seen to be highly expressed during sporulation, being the most important synthase at this stage. Surprisingly, synthesis of α 1,3 glucan was affected by two amylases, one which stimulated, whereas the other inhibited α 1,3 glucan synthesis (He et al., 2014).

The mechanism of pullulan synthesis has not been deeply explored, and its mechanism remains mostly unknown. It was described that the polysaccharide is synthesized intracellularly in *A. pullulans*, and then secreted (Simon et al., 1998). Summarizing previous observations from Catley and McDowell, 1982, LeDuy et al., 1988, and Cheng et al. (2011), it may be concluded that the polysaccharide is synthesized from UDPGlc with the involvement of a lipid phosphate that accepts two succesive glucosyl moities from UDPGlc to form an isomaltose molecule (Lipid-P-Glc-α-1-6-Glc), finally accepting a further glucosyl unit from UDPG to form an isopanosyl intermediate (Lipid-P-Glc-α-1-6-Glc-α-1-4 Glc). These isopanosyl moities are further on polymerized to form the final pullulan polysaccharide.

### 3.5 Phylogenetic analysis of fungal alpha 1,3 glucan synthases

Search of genes encoding α 1,3 glucan synthases already annotated in the fungal species whose genes have been sequenced, or that we identified by homology in NCBI and JGI, revealed that they are present exclusively in Dikarya: Ascomycota and Basidiomycota.

These genes contain an open-reading frame made of an average of 2376 ± 166 amino acids. (309 entries).

By use of MEME (http://meme-suite.org/info/) (Bailey and Elkan (1994), PFAM and NCBI, we identified important conserved motifs in 309 α 1,3 glucan synthases. These were: *i*) DIEAFGVFPDWQRQLAKFASVQDRLREWHPSVREKJIRFSCMIIASLDID, corresponding to alpha amylase catalytic domain found in α 1,3-glucan synthase (also called uridine diphosphoglucose-1,3-alpha-glucan glucosyltransferase, and 1,3-d-glucan synthase with an E-value of 1e-^24^, and an identity of 92%, ii) WQTHGCYSLGSSQYYNWPJEKGRTGCHDDTVSYDHRDPSHPVRNIIKHMY,

corresponding to alpha amylase catalytic domain also found in α 1,3-glucan synthase with an E-value of 3e^−23^ and an identity of 78%; and iii) QHFGTIQDWRDAITEIHKRGMYVJFDNTVATMGDLIGFEGYLNTSTPFSL, alpha amylase catalytic domain found in α 1,3-glucan synthase, with an E-value of 2e^−23^, and an iddentity of 88%. These data suggest that α 1,3 glucan synthases probably descends from another alpha transglycosidase in an ancestor of Dikarya, and agree with a preliminary hypothesis obtained with the analysis of a fewer number of sequences (Ruiz-Herrera, 2012).

In Ascomycota, the groups that contain more species with α 1,3 glucan synthases are Eurotiomycetes, Saccharomycetes and Chaetotryomycetes, in that order of abundance.

In the case of Basidiomycota, whose genomes have been sequenced thus far, a high number of species of Puccinomycotina and Agaricomycotina that have been sequenced were found to contain one or several genes encoding these enzymes. On the other hand, only in two species of Ustilaginomycotina, *Meira miltonrushii* and *Exobasidium vaccii* (Exobasidiomycetes; Exobasidiales; Brachybasidiaceae) genes with 60% and 44% respective homology to Ascomycota α 1,3 glucan synthases were identified.

One interesting observation is that in general, within the same taxonomic group, and even in the same genera there are species that contain or not genes encoding the synthase. These results suggest that after acquisition of the gene(s) encoding α 1,3 glucan synthases, this trait was either conserved or lost along evolution of the different species. The high homology of α 1,3 glucan synthases present in the different species speaks against, but of course does not rule out, that the gene was acquired by the different taxa at different periods of evolution.

**Fig. 1:**
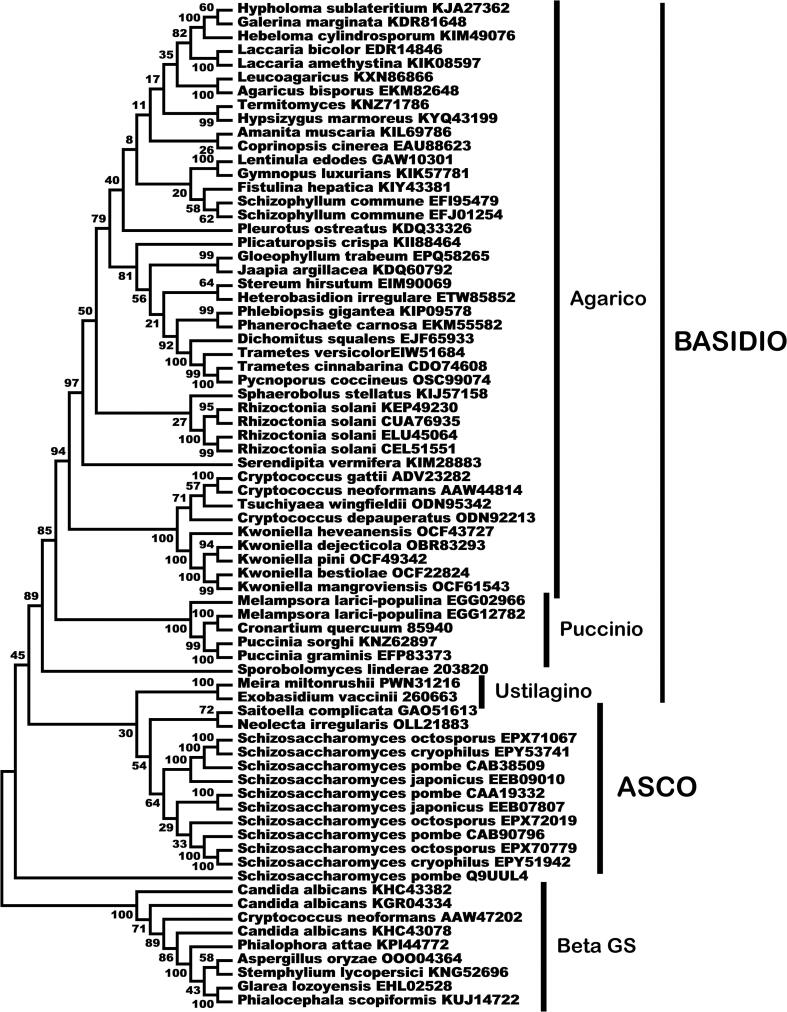
Evolutionary relationships of alpha glucan synthases from the Basidiomycota subphyla in relation to the enzymes from Ascomycota. The MEGA6 program (Tamura et al., 2013) was used to obtain the dendrogram generated by the Maximum Likelihood method based on the Le Gascuel 2008 model (Le and Gascuel, 1993), with 1000 bootstraps (Felsenstein, 1985). The percentage of trees in which the associated taxa clustered together is shown next to the branches. Initial tree(s) for the heuristic search were obtained by applying the Neighbor-Joining method to a matrix of pairwise distances estimated using a JTT model. A discrete Gamma distribution was used to model evolutionary rate differences among sites [5 categories (+*G*, parameter = 0.8908]. Evolutionary analyses were conducted in MEGA6 (Tamura et al., 2013). BASIDIO, Basidiomycota; ASCO, Ascomycota; Agarico, Agaricomycotina; Puccinio, Pucciniomycotina; Ustilagino, Ustilaginomycotina. Beta GS, rooting group of β-1,3 glucan synthases.

**Fig. 2:**
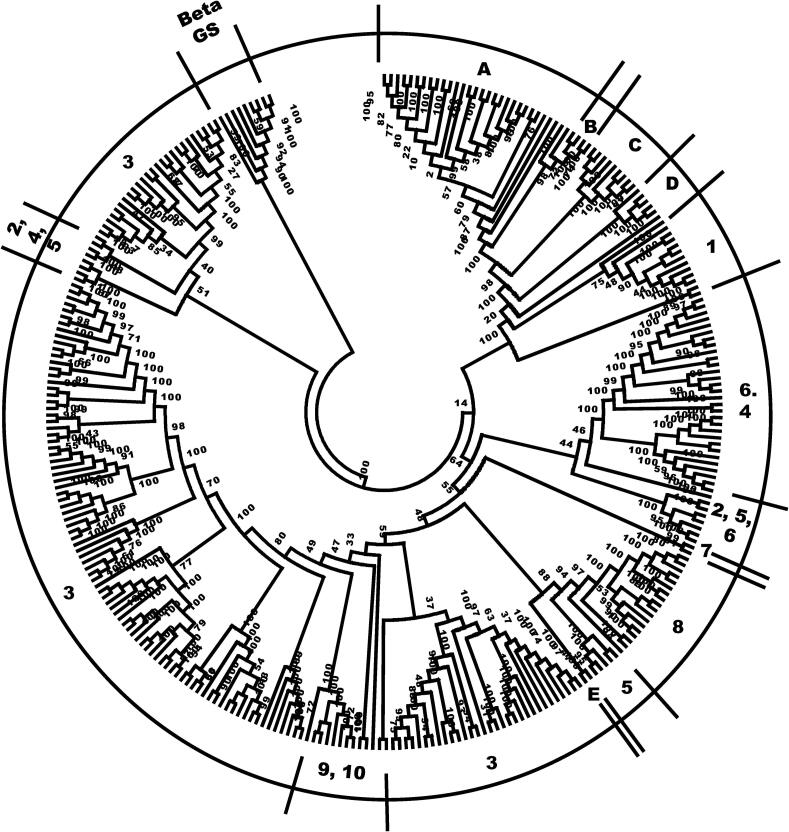
Phylogenetic tree showing the relationships of the identified fungal α 1,3 glucan synthases. Analysis was made of 309 sequences as described for Fig. 1, with 100 bootstraps. Letters refer to orders of Basidiomycota: A, Agaricales, Polyporales, Gloeophyllales, Jaapiales, Russulales, Amylocorticiales, Sebacinales, and Geastrales, B, Cantharellales, C, Tremellales, D, Pucciniales and Spiculogloeales, and E, Exobasidiales. Numbers refer to orders of Ascomycota: 1, Schizosaccharomycetales, Taphrinales, and Neolectales, 2, Dothideales, 3, Eurotiales, 4. Sordariales and Botryosphaeriales, 5, Capnodiales, 6, Glomerellales, Xylariales, Diaporthales, Coniochaetales, Pleosporales, Hypocreales and Magnaporthales, 7, Helotiales, 8. Chaetothyriales, 9, Verrucariales and Phaeomoniellales, and 10, Onygenales. Beta GS, β 1, 3 glucan synthases rooting group. Letters and numbers correspond to the fungal orders involving the species described in Table 1, that were used to construct the tree.

A phylogenetic analysis of the available sequences of α 1,3 glucan synthases revealed that, as previously shown with a limited number of sequences (Ruiz-Herrera, 2012), the enzymes belonging to Basidiomycota and Ascomycota formed two separate clades (Fig. 1, Fig. 2). Interestingly, the closest similarities to the Basidiomycota enzymes are those from *S. pombe*. In Fig. 2 we marked the branches of the phylogenetic tree that correspond to distinct clades of the Ascomycota that contain genes encoding α 1,3 glucan synthases (the enzymes and fungal species used to contruct the tree are described in Table 1).

**Table 1:** Species of Dikarya where α 1;3 glucans synthases have been identified and were used to construct the phylogenetic tree of Fig. 2.

***Basidiomycota***
**A. Agaricales:***Agaricus bisporus; Amanita muscaria; Coprinopsis cinérea; Fistulina hepatica; Galerina marginata; Hypholoma sublateritium; Hebeloma cylindrosporum; Hypsizygus marmoreus; Laccaria amethystina; L. bicolor; Lentinula edodes; Leucoagaricus sp; Pleurotus ostreatus; Schizophyllum commune; Termitomyces* sp.
**Polyporales:***Dichomitus squalens; Phanerochaete carnosa; Phlebiopsis gigantea; Pycnoporus coccineus; Trametes cinnabarina; T. versicolor*
**Gloeophyllales:***Gloeophyllum trabeum*
**Jaapiales:***Jaapia argillacea*
**Russulales:***Heterobasidion irregulare; Stereum hirsutum*
**Amylocorticiales:***Plicaturopsis crispa*
**Sebacinales:***Serendipita vermifera*
**Geastrales:***Sphaerobolus stellatus*
**B. Cantharellales:***Rhizoctonia solani*
**C. Tremellales:***Kwoniella heveanensis; K. pini; K. dejecticola; K. mangroviensis; K. bestiolae; Tsuchiyaea wingfieldii; Cryptococcus depauperatus; C.gattii; C. neoforman.*
**D. Pucciniales:***Cronartium quercuum; Melampsora larici-populina; Puccinia graminis; P. sorghi*
**Spiculogloeales:***Sporobolomyces linderae*
**E. Exobasidiales:***Exobasidium vaccinii; Meira miltonrushii*

***Ascomycota***
**1. Schizosaccharomycetales**: *Schizosaccharomyces cryophylus; S. japonicus; S. octosporus; S. pombe*
**Tapthynales**: *Saitoella complicada*
**Neolectales**: *Neolecta irregularis*
**2. Dothideales:***Aureobasidium melanogenum; A. namibiae; A. pullulans*
**3. Eurotiales:***Aspergillus aculeatus; A. arachidicola; A. calidoustus; A. carbonarius; A. clavatus; A. flavus; A. fumigatus; A. kawachii; A. lentulus; A. luchuensis; A. nidulans; A. niger; A. nomius; A. ochraceoroseus; A. oryzae; A. rambellii; A. ruber; A. terreus; A. thermomutatus; A. turcosus; A. udagawae; Byssochlamys spectabilis; Elaphomyces granulatus; Penicillium antarcticus; P. brasilianum; P. camemberti; P. chrysogenum; P. coprophilum; P. decumbens; P. digitatum; P. flavigenum; P. freii; P. griseofulvum; P. italicum; P. nalgiovense; P. nordicum; P. oxalicum; P. polonicum; P. roqueforti; P. rubens; P. solitum; P. steckii; P. vulpinum; Rasamsonia emersonii; Talaromyces atroroseus; T. cellulolyticus; T. cinnabarina; T. islandicus; T. marneffei; T. stipitatus; T. versicolor*
**4. Sordariales:***Neurospora crassa; N. tetrasperma; Podospora anserina; Sordaria macrospora*
**Botryosphaeriales:***Neofusicoccum parvum*
**5. Capnodiales:***Mycosphaerella eumusae****;****Pseudocercospora fijiensis; P. musae, Zymoseptoria brevis; Z. tritici*
**6. Glomerellales:***Colletotrichum chlorophyti; C. fioriniae; C. graminicola; C. gloeosporioides; C. higginsianum; C. nymphaeae; C. orbiculare; C. orchidophilum; C. salicis; C. simmondsii; C. tofieldiae*
**Xylariales:***Pseudomassariella vexata*
**Diaporthales:***Diapothe helianthe, D. ampelina, Valsa mali*
**Coniochaetales:***Coniochaeta ligniaria*
**Pleosporales:***Clohesyomyces aquaticus; Stagonospora* sp.
**Hypocreales:***Fusarium oxysporum*
**Magnaporthales:***Magnaporthe oryzae*
**7. Helotiales:***Botrytis cinerea, Sclerotinia borealis*
**8. Chaetothyriales:***Cladophialophora bantiana; C. carrionii; C. immunda; C. psammophila; C. yegresii; Capronia epimyces, C. coronata, Cyphellophora europea; Exophiala aquamarina; E. mesophila; E. oligosperma; E. sideris; E. spinifera; E. xenobiotica; Fonsecaea pedrosoi; Phialophora americana; Rhinocladiella mackenziei*
**9. Verrucariales:***Endocarpon pusillum*
**Phaeomoniellales:***Phaeomoniella chlamydospora*
**10. Onygenales:***Blastomyces dermatitidis, B. gilchristii, Coccidioides immitis; C. posadasii; Emmonsia crescens, E. parva, Histoplasma capsulatum, Paracoccidioides brasiliensis*

One inconsistency found was that the enzymes belonging to Ustilaginales, did not appear within the rest of α 1,3 glucan synthases from Basidiomycota, but among the Ascomycota orders of Capnodiales and Chaetothyriales (Fig. 2). No explanation exists for this anomalous result.

Another interesting result is that some fungi belonging to the same order may have enzymes belonging to different classes. Examples of this are groups 2, 3 and 5 of Fig. 2. This result agrees with our suggestion regarding to the acquisition and loss of alpha glucan synthases along evolution (see above)

## 4 Fungal wall beta glucans

### 4.1 Types and distribution of beta glucans

As it was indicated above, the different types of beta glucans existing in fungi were early revised by Ruiz-Herrera (1992). Nevertheless, the most thorough taxonomical description of fungal beta glucans based on their chemical structure, is the review made by Synytsya and Novák (2013). Similarly to alpha glucans, the authors divided beta glucans in groups according to their structure. The first group (*i*) was composed by linear β d-glucans, divided into several subgroups; one containing linear β-glucans having a single type of glycosidic linkage (*i.i*). The most abundant glucans of this type are β 1,3 glucans present in many fungal species, where they constitute a basic structure of the cell wall (see Ruiz-Herrera, 1992, Ruiz-Herrera, 2012). Probably the most studied β 1,3 glucans are those from *S. cerevisiae*, followed by a great number of reports describing its existence in a large number of species. Another glucan belonging to this subgroup is pustulan, a polysaccharide made of glucosyl units joined through β 1,6 linkages, initially identified in lichens, but also described in *S. cerevisiae*, and further on in a large number of species (summarized by Synytsya and Novák, 2013). The second subgroup of linear beta glucans according to these authors, (*i.ii*), is made by linear glucans containing more than one type of glycosidic linkage. Among these, the authors cite lichenan, a polysaccharide having both β 1,3 and β 1,4 linkages, that as its name suggests, has been identified in a number of lichens.

The second group of beta glucans described by Synytsya and Novák (2013) (*ii*), includes branched beta glucans. Of these, the most abundant, and present in the largest number of fungal species analyzed are branched β 1,3 glucans. Most of these contain β 1,6 branches of different size and characteristics (*ii.i*), the first one of them described in *S. cerevisiae* (Ruiz-Herrera, 1991, Ruiz-Herrera, 1992, see above). Synytsya and Novák (2013) notice that some of these polysaccharide have received specific names, among them: grifolan, extracted from *Grifola frondosa*; schizophylan, isolated from *Schizophylum commune*; scleroglucan from *Sclerotium sp*, and botryosphaeran from *Botryosphaeria sp*. A polysaccharide made of a chain of glucosyl units linked by β 1,3 bonds with β 1,4-linked branches is calocyban from the basidiocarps of *Calocybe indica*. The second subgroup (*ii.ii*), according to the multicited authors, is made of branched β 1,6 chains. A polysaccharide of this type containing a branch made solely of single β 1,3-linked glucosyl moiety was found in *Pleurotus florida* and *Lentinula edodes.* Other polysaccharides containing short β 1,3 branches of β 1,3-linked glucosyl moieties were described in *Agaricus brasiliensis* and *C. albicans*, whereas in a polysaccharide from *Sarcodon aspratus* the branches are longer.

The other subgroup), (*ii.iii*), contains the branched beta glucans with more than one type of linkage. Among them, the authors cite pleuran form *Lentinula* (*Lentinus*) *squarrosulu*s that contains a glucan chain containing both β 1,3 and β 1,6 bonds, and β 1,6 bound branches made of a single glucose molecule. In a similar glucan from *Phellinus ribis*, the chain contains also β 1,4 linkages (description made by cited authors).

The final group (*iii*) was the one made by glucans containing both β and α bonds that was divided into two subgroups: (*iii.i*) made of linear polysaccharides, and (*iii.ii*) containing branched glucans. In the first subgroup, a glucan from the fruiting bodies of *Astraeus hygrometricus* made by glycosyl moieties alternatively bound by α 1,4 and β 1,6 bonds was included, whereas in another glucan from *Termitomyces microcarpus* the glucosyl moities are bound by alternating α 1,4 and β 1,3 linkages. Regarding the branched polysaccharides of the second subgroup, these were separated into several minor groups: (*iii.ii.i*) made of glucans containing an alpha chain joined to a beta branch. Among these, the structure of a glucan from *Piptoporus betulinus* is made of α 1,3 chains with a branch made of a single glucose unit bound by an β 1,6 linkage, whereas in a glucan from *P. florida* the β 1,6 linked branch was made of a β 1,3 linked glucose chain. In the second minor group (*iii.ii.ii*), glucans containing β-bound chains with α–bound branches, the authors cite a soluble polysaccharide isolated from basidiocarps of *Calocibe indica*. In the third minor (*iii.ii.iii*) group made by glucose polysaccharides joined by both α and β chains and α linked branches, glucans from the fruiting bodies of *P. florida* and *Calocibe indica* were included. And finally, within the fourth minor group (*iii.ii.iv*), the authors included polysaccharides having glucan chains with α and β linkages, and β linked branches. Among them, glucans present in *Pleutotus sacor-caju*, and *Volvariella diplasia* were cited by the authors.

Independently of the large variations in their substitutions shown above, the most important and abundant polysaccharides of the fungal cell walls are β 1,3 glucans, whose amounts have been calculated to be as much as 65–90% of the total wall glucans (Bowman and Free, 2006). β 1,3 Glucans have been found in practically all the fungal phyla, with the probable exception of Microsporidia. The case of Chitridiomycota is peculiar. In the literature, there is a single description of the chemical composition of the mycelium cell wall of a Chitridiomycota species, *Allomyces macrogynus* (Aronson and Machlis, 1959), where it was described the presence of a glucan, possibly (but not confirmed) a β 1,3 glucan. Accordingly, the subphylum Chitridiomycota was included in the group of fungi with a wall made of chitin and glucan in the classic descriptions of Bartnicki-García (1968), and by Ruiz-Herrera (1992). The identification of genes encoding β 1,3 glucan synthases in several species of Chtridiomycota (see below) supports this concept.

### 4.2 Structure of beta 1,3 glucans

β 1,3 glucans are probably the most important and abundant polysaccharides of the fungal cell walls [their amounts have been calculated to be as much as 65–90% of the total wall glucans (Bowman and Free, 2006)]. The earliest studies on the structure of fungal glucans were made with those secreted by *S. cerevisiae* secreted during protoplast regeneration. This glucan appeared in the form of microfibrils roughly 4 μm in length and 20 nm in diameter (Kreger and Kopecká, 1975, Kopecká and Kreger, 1986). This microfibrilar structure was also observed in β 1,3 glucans synthesized *in vitro* by a soluble fraction obtained from yeast cells (Larriba et al., 1981). Bluhm and Sarko (1977), through their analysis of lentinan, a β 1,3 glucan from *L. edodes*, suggested that linear fungal β 1,3 glucans were made by the association of three molecules of the polysaccharide, associated by hydrogen bonding, a result that was later on supported by the studies of Kopecká and Kreger (1986) on the polysaccharide from *S. cerevisiae*. Nevertheless, Saito et al. (1987) analyzed the structure of a number of fungal branched β 1,3 glucans and confirmed their triple-helix form, with the exception of lentinan that corresponded, according to the authors, to a single chain helix, that only after liophilization (*i.e.* desiccation) acquired the triple-helix structure.

However, as indicated above, the existence of linear β 1,3 glucans is not the rule, since most of them contain different branches mostly joined by β 1,6 bonds. This was known since the earliest studies of Manners et al. (1973), and later on extended to a large number of fungal species. Apparently, the formation of linear β 1,3 glucans is a rare event occurring under peculiar conditions, for example during protoplast regeneration (Kreger and Kopecká, 1975, Kopecká and Kreger, 1986), or by soluble extracts of *S. cerevisiae* (Larriba et al., 1981). Also, we described the synthesis of an unbranched β 1,3 glucan by a mutant of *Ustilago maydis* deficient in the transcription factor PacC/Rim101 of the Pal/Rim pathway involved in the control of pH (Fonseca-García et al., 2011). On the contrary, β 1,3 glucans synthesized *in vitro* by membrane fractions of *S. cerevisiae* were branched. This was determined by exhaustive digestion with an exo β 1,3 glucanase that left besides glucose, intact gentiobiose and short oligosaccharides (López-Romero and Ruiz-Herrera, 1977).

### 4.3 The roles of beta glucans

Considering the microfibrilar structure of the most abundant β 1,3 glucans, it is widely accepted that they have a role in the architecture and resistance of the cell wall, although other roles cannot be dismissed (*e.g.* Latgé, 2007, Ruiz-Herrera, 2012). Their importance in cell wall structure is revealed by different studies demonstrating that enzymatic digestion or inhibition in the synthesis of β 1,3 glucans, leads to either cell lysis or alterations in their morphology. For example, Miyata et al. (1985) demonstrated in *S. pombe* that inhibition of β 1,3 glucan synthesis by aculeacin, or its digestion by β 1,3 glucanase, led to cell autolysis or loss of the normal morphology of the cells. These appeared dumb-belled or like round flasks. It is also a common observation that protoplasts of *S. cerevisiae* can be obtained by treatment with β 1,3 glucanase (the amounts of chitin, the other structural component of the yeast wall, are very low). In this sense the results obtained by Liu et al., 1999, Cortés et al., 2007 are significant. These authors observed that *S. pombe* mutants in a gene encoding a β 1,3 glucan synthase subunit were unable to make the cell septum. Moreover, studies on the localization of a β 1,3 glucan synthase subunit in *S. pombe* cells suggested that it was involved not only in septation, but also in mating, and spore formation and germination (Cortés et al., 2002, Cortés et al., 2007). These data were in line with the identification of a β 1,3 glucan synthase specifically involved in the maturation of the ascospore wall of this fungus (Martín et al., 2000). Other observations in the same direction revealed that homologous forms of β 1,3 glucan synthase were involved in the assembly of the *S. cerevisiae* spore wall (Ishihara et al., 2007).

One important aspect of fungal beta glucans is their effect on mammals, man included, and on plants. Great interest has developed recently about the mechanisms of recognition of beta glucans in animals, and the literature on this subject is copious (for a review see Brown and Gordon, 2003). Thus, beta glucan molecules can be recognized by both vertebrate and invertebrates, and are considered to be pathogen-associated molecular patterns (PAMPs). The development of a recognition system of β glucans by these animals appears to be a mechanism of resistance to fungal infections. In vertebrates they are recognized by membrane receptors, and in invertebrates by receptors located in the hemolymph (Brown and Gordon, 2003). In both systems, beta glucans induce activation of the immune system that in vertebrates, human included, may depend on the nature and molecular size of the polysaccharide. Interestingly, in general their medical application has been considered to be helpful in the treatment of different diseases, including cancer, and infection by fungi, bacteria, protozoa and viruses (see review by Chen and Seviour, 2007). Recognition of these polysaccharides involves a great number of receptors including Toll-like receptors, CR3, scavenger receptors, LacCer and TLR (see Chen and Seviour, 2007), but the main receptor of beta glucans is now considered to be dectin-1, a type II transmembrane C-type lectin-like component located in the macrophages. Use of mice mutants deficient in this receptor demonstrated its role in fungal resistance. Accordingly, these mutant mice proved to be extremely sensitive to infections by *C. albicans,* and showed an impairement in the inflamatory response measured by macrophage TNF production to both *C. albicans* and *Aspergillus fumigatus* (Taylor et al., 2007).

Interestingly, not only animals but also plants have mechanisms for detection of β glucans that they “interpret” as a danger signal, therefore behaving in them also as PAMPs (see review by Fesel and Zuccaro, 2016). The mechanism of reception in plants is elusive by several aspects, mainly perhaps because plants also posses β 1,3 glucans. Nevertheless, in *Arabidopsis thaliana*, a dectin-1 homologue was identified with an identity of about 30% to the mammalian receptor. Also it may be cited that Melida et al (2018) described that β 1,3 glucan oligosaccharides triggered immune responses in *A. thaliana*. Accordingly, the possibility that other receptors of glucans exist in plants is an open question. Apparently not only β 1,3 glucans, but also only β 1,6 glucans have a role in this function, at least in maize plants, according to data described by Oliveira-Garcia and Deising (2016).

### 4.4 Synthesis of beta glucans

#### 4.4.1 Introduction

Those authors interested on the early literature on the synthesis of fungal beta glucans may get a glimpse in the review by Ruiz-Herrera (1991). One problem regarding the analysis of the synthesis of these polysaccharides is their extreme variety as described above. In this sense it may be indicated that the beta glucan whose synthesis has been studied to a deeper level are β 1,3 glucans. Lagging behind this, some data exist on the mechanism involved in the synthesis of β 1,6 glucans, whereas some interesting information has been gained more recently on some enzymes that appear to play a role in glucan branching (see below).

#### 4.4.2 Synthesis of β 1,3 glucans

Initial analysis on the synthesis of β 1,3 glucans utilized *S. cerevisiae* as subject of study, using whole cells and crude membranes as the enzyme source. The first succeful experiments that obtained the *in vitro* synthesis of β 1,3 glucan were reported by Sentandreu et al. (1975), and Lopez-Romero and Ruiz-Herrera, 1977). Previous unsuccessful trials were apparently due to the high instability of the enzyme in crude extracts. Further experiments with high-speed supernatants of *S. cerevisiae* extracts demonstrated that the synthesized product appeared in the form of microfibrils (Larriba et al., 1981).

It has been demonstrated that all β 1,3 glucan synthases from the different fungal species use UDPglucose (UDPGlc) as the only glucosyl donor, having a high Km for the substrate. It is also known that they are not activated *in vitro* by controlled proteolysis, as occurs with chitin synthases, and that they are activated or not (depending on the system) by divalent cations. In addition, their optimal pH is in the neutrality zone, and their optimal activity occurs around room temperature. The most characteristic inhibitors of β 1,3 glucan synthases are the members of two families of antibiotics that paradoxically are produced by fungi: papulacandins produced by strains of *Papularia sphaerosperma*, and echinocandins synthesized by *Aspergillus regulovalvus.* These compounds were found to inhibit the growth of different fungi, mainly human pathogens. Later on their mode of action was pinpointed to their inhibitory effect on β 1,3 glucan synthases, that occurs by a non-competitive mechanism. Considering the importance of β 1,3 glucans in the structure of the cell wall, and their abundance in fungal species, these compounds, and some synthetic derivatives of them, have become the drugs of choice for the treatment of mycoses, mainly profound mycoses that have become an important medical problem (see reviews by Debono and Gordee, 1994, Douglas, 2001, Gupta and Thomas, 2003). More recently, other inhibitors of β 1,3 glucan synthases have been described. Among these, poacic acid (a natural product) was found to inhibit the enzyme in a manner dependent of the calcineurin pathway (Lee et al., 2018). Interestingly, susceptibility varied among the *Candida* species analyzed, and did not correspond to the sensitivity to caspofungin, suggesting that their modes of action are different. Enfumafungin, a glycoside triterpene produced by *Hormonema* sp. was also found to be a specific inhibitor of glucan biosynthesis *in vivo* and *in vitro* (Schwartz et al., 2000).

Regarding the catalytic mechanism of β 1,3 glucan synthase, it has been demonstrated that there is no lipid intermediate involved in the synthesis of the polysaccharide, as occurs with some other glucosyl transferases. On the other hand, it has not been resolved whether there is or not an acceptor for the intiation of the glucan chain molecule. In this direction it was demonstrated that yeast β 1,3 glucan microfibrils synthesized *in vitro* lacked a free reducing end (Larriba et al., 1981). In addition, Andaluz et al., 1986, Andaluz et al., 1988) observed that *in vitro* synthesized β 1,3 glucan by *S. cerevisiae* and *C. albicans* was partially extracted with hot SDS (an anomalous behavior of beta-joined polysaccharides), and was susceptible to digestion by both, a beta glucanase and a protease. Ruiz-Herrera and Larriba (1995), making pulse and chase experiments of β 1,3 glucan synthesis in *S. cerevisiae* membrane fractions, observed that the SDS-soluble glucan product served as an acceptor for the synthesis of insoluble high MW β 1,3 glucan. All these results suggested the existence of an acceptor involved in the initiation of the synthesis of β 1,3 glucan, probably of proteinaceous nature. Unfortunately, no further studies on the subject have followed.

One important observation for the understanding of the organization of β 1,3 glucan synthase was the demonstration that the active complex of the enzyme is made of two components, one that remained membrane-bound, later on identified as the active protein (see below), and the other, an activator protein identified as Rho1, a small GTP-binding protein (Mazur and Baginski, 1996, Qadota et al., 1996). Enzymatic activity of the glucan synthase required binding of Rho1 (Mazur and Baginski, 1996) to the membrane bound protein. However Rho1 is not specific for glucan synthesis, since it has been demonstrated to be involved in several additional functions (see review by Douglas, 2001). Although in the former literature it was insisted that glucan synthase was a protein of the plasma membrane, further data demonstrated that it arrives to the membrane in specific vesicles (Riquelme, 2013). This situation is logical, since no proteins are synthesized at the plasma membrane; they are synthesized in the endomembrane system of the cell, and later on they are transported by vesicles and microvesicles to different organelles. Indeed, it has been demonstrated that the catalytic protein of glucan synthase and Rho1 are transported by different vesicles to the plasma membrane, where activation of the enzyme and catalysis occur (Verdin et al., 2009, Riquelme, 2013). The transport of these vesicles most likely involves members of the cytoskeleton. Thus, Utsugi et al. (2002) observed that GFP-labelled Fks1p (β 1,3 glucan synthase) was associated to actin patches that located at the sites of wall growth in *S. cerevisiae*.

#### 4.4.3 Genes encoding β 1,3 glucan synthases.

Identification of the gene(s) coding for β 1,3 glucan synthase involved different approaches, some of them completely unrelated to the function of the encoded product. In fact, the designation of the genes: *FKS*, has absolutely no indication of the gene function, since it corresponded to hypersensitivity to the immunosuppressive agent FK506 by *S. cerevisiae* mutants (Parent et al., 1993, Eng et al., 1994). Other means to identify the genes encoding β 1,3 glucan synthase were the use of mutants showing phenotypes more closely related to cell wall alterations. Among these we may cite, sensitivity to calcofluor white, association to the β 1,3 glucan synthase protein, and sensitivity to inhibitors of β 1,3 glucan synthesis. That all these characteristics corresponded to the gene that encodes β 1,3 glucan synthase was further on clarified by Douglas et al. (1994) (see review by Douglas, 2001).

Later studies demonstrated the existence of homologs of the *S. cerevisiae FKS* genes (also called *BGS* genes), and *RHO* genes in a number of fungal species, but one interesting observation is that *FKS* homologs are absent in Microsporidia. In Chtridiomycota the presence of genes *KRE6* and *SKN1*, putatively implicated in β 1,6 glucan synthesis, was observed (see below), and the presence of a glucan tentatively identified as β 1,3 glucan was found in the cell wall of a Chitridiomycota species (see above). The lack of a definite identification of β 1,3 glucans, and of *FKS* (*BGS*) genes in the species analyzed in this group of fungi up to 2010, made us suggest that the genes encoding β 1,3 glucan synthases appeared when Zygomycota departed from the fungal evolution line (Ruiz-Herrera and Ortiz-Castellanos, 2010). However, sequencing of the genomes of a larger number of Chitridiomycota species, allowed us to demonstrate by homology analysis, the presence of *FKS* (*BGS*) gene homologs in *A. macrogynus* and *Blastocladiella britannica*, altough other species of the phylum did not have any homolog gene. Accordingly, the previous hypothesis must be changed now to indicate suggest that β 1,3 glucans appeared for the first time in this fungal group.

In this sense, it is interesting to note that *FKS* genes have a high homology to those encoding β 1,3 glucan synthases from plants, algae and Chromista. All of them fall into the family 48 of glycosyltransferases, and lack the characteristic motif associated to activity in other processive beta polysaccharide synthases, such as chitin and cellulose synthases: QXXRW. Accordingly, we suggested that *FKS* genes were possibly horizontally transferred to fungi from a member of the Plant or Chromista kingdoms (Ruiz-Herrera and Ortiz-Castellanos, 2010).

One interesting observation is that some fungal species contain more than one homolog of the initially identified *S. cerevisiae FKS* gene, denominated *FKS1*. Accordingly, they may contain 2 to 4 gene homologs. In some fungi, different regulation and functions have been described for the several genes. Probably the best studied species is *S. cerevisiae* where three *FKS* genes were identified: *FKS1* and *FKS2* with high homology, and *FKS3* with a lower homology. Regulation of genes *FKS1* and *FKS2* was different, and while mutation of only *FKS1* reduced the levels of β 1,3 glucan, mutation of both *FKS1* and *FKS2* genes was lethal, indicating that both of them are essential (Mazur et al., 1995). Later data showed that Fks1 and Fks2 synthases are important for the assembly of the ascospore wall (Ishihara et al., 2007). In the case of *S. pombe*, four genes homologs to *FKS1* (*BGS1* to *4*) have been identified, and mutation of one of them (*CPS1*, later on named *BGS1*), demonstrated that it was not involved in cell growth, but specifically in septum formation (Liu et al., 1999). *BGS2* was found to be involved in ascospore wall synthesis (Liu et al., 2000), and *BGS3* and *BGS4* in the synthesis of the apical wall. Contrasting with these data, the only *FKS1* homolog of the maize pathogen *U. maydis* was constitutively expressed both *in vitro* and *in planta* (Robledo-Briones and Ruiz-Herrera, 2012). In other mycelial fungi having only one *FKS* gene, this is essential (see Thompson et al., 1999, Ha et al., 2006, Riquelme, 2013).

### 4.5 Phylogenetic analysis of fungal beta 1,3 glucan synthases.

As described above for α 1,3 glucans, use of MEME (http://meme-suite.org/info/), PFAM and NCBI for the analysis of 509 sequences, permited the identification of different conserved motifs, classified as belonging to the β1,3 glucan synthase family CL0111. Those showing the highest homology were the following: *i*) KAQKGLHLNEDIYAGMNALLRGGRIKHCEYYQCGKGRDLGFGSILNFTTK, with an E-value of 2e^−31^; *ii*) *ENIGILGDVAAGKEQTFGTLFARTLAQIGGKLHYGHPDFLNGIFMTTRGG*, with an E-value of 1.2 e-28; and *iii*) SGNPILGDGKSDNQNHAIIFYRGEYIQLIDANQDNYLEECL, with an E-value of 7.4 e^−26^.

**Fig. 3:**
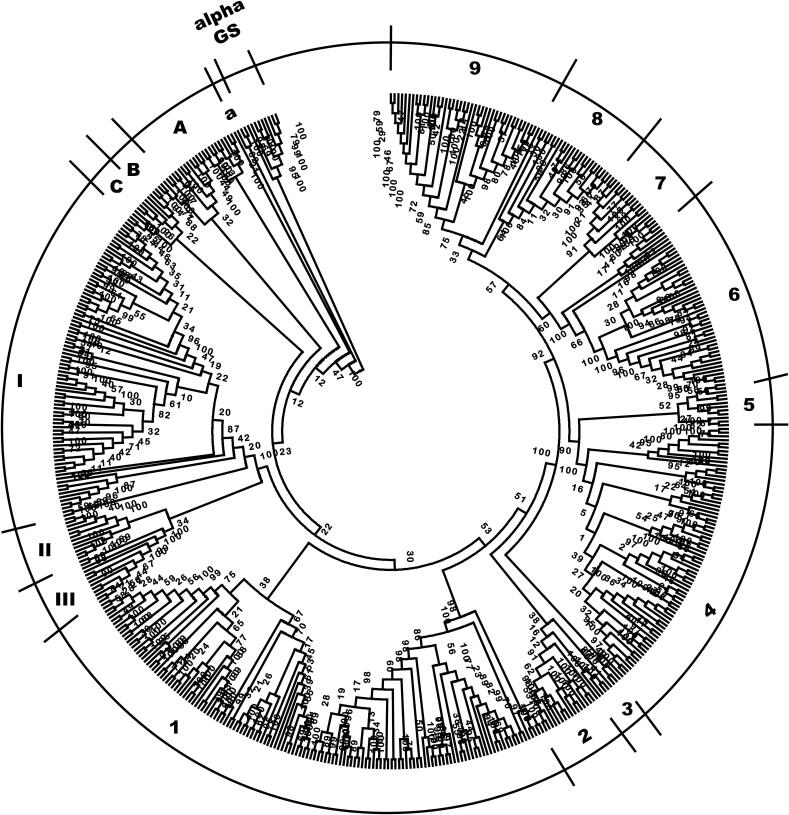
Phylogenetic tree showing the relationships of identified fungal β 1, 3 glucan synthases. Analysis of 500 sequences was made as described for Fig. 1, with 100 bootstraps. Lower case letter a refers to Chitridiomycota of the order Blastocladiales. Capital letters refer to orders of Mucoromycota: A, Mucorales, B, Entomophthorales, Kickxellales, and Harpellales, C, Basidiobolales and Mortierellales. Roman numerals refer to orders of Basidiomycota: I, Agaricales, Boletales, Corticiales, Polyporales, Russulales, Gloeophyllales, Jaapiales, Hymenochaetaleas, Sebacinales, Cantharellales, Dacrymycetales, Geastrales, Auriculariales, Amylocorticiales, Tremellales, Trichosporonales, and Wallemiales, II, Pucciniales, Mixiales, Microbotryales, Leucosporidiales, and Sporidiobolales, III, Ustilaginales, Ceraceosorales, and Malasseziales. Arabic numerals refer to orders of Ascomycota, 1, Saccharomycetales, 2, Schizosaccharomycetales, 3, Taphrinales, Neolectales, Pneumocystidales, Pezizales, and Orbiliales, 4, Hypocreales, Microascales, Glomerellales, Magnaporthales, Diaporthales, Ophiostomatales, Sordariales, Xylariales, and Togniniales, 5, Helotiales, and Erysiphales, 6, Eurotiales, 7, Onygenales, 8, Chaetothyriomycetales, Umbilicariales, Verrucariales, 9, Capnodiales, Dothideales, Venturiales, Botryosphaeriales, and Pleosporales. alpha GS, α 1, 3 glucan syntases rooting group. Letters and numbers correspond to the fungal orders involving the species described in Table 2, that were used to construct the tree.

As it was described for α 1,3 glucan synthases, the phylogenetic analysis of β 1,3 glucan synthases, showed that the enzymes belonging to the phyla (in this case, 4) with species containing these enzymes: Chitridiomycota, Zygomycota, Ascomycota and Basidiomycota, formed separate groups. The data obtained indicate that the phylogenetic relationships of the different enzymes belonging to the four phyla formed separate clades corresponding to single or mixtures of fungal orders, that are designated in the phylogenetic tree by lower letters, capital letters, roman numbers, and Arabic numbers respectively (Fig. 3; the species used to construct the tree are shown in Table 2). In contrast to alpha 1,3 glucan synthases, it was observed that the species of the same order contain 1,3 beta glucan synthases of the same class.

**Table 2:** Species of Fungi where β 1;3 glucan synthases have been identified and were used to construct the phylogenetic tree of Fig. 3.

***Chitridiomycota***	
**a.**	**Blastocladiales:***Allomyces macrogynus, Blastocladiella britannica*
	***Mucoromycota***
**A.**	**Mucorales:***Absidia repens, Hesseltinella vesiculosa, Lichtheimia corymbifera, L. ramosa, Mucor ambiguus, M. circinelloides, Phycomyces blakesleeanus, Rhizopus delemar, R. microsporus, Syncephalastrum racemosum*
**B.**	**Entomophthorales:***Conidiobolus coronatus*
	**Kickxellales:***Linderina pennispora*
	**Harpellales:***Smittium culicis, S. mucronatum*
**C.**	**Basidiobolales:***Basidiobolus meristosporus*
	**Mortierellales:***Lobosporangium transversale, Mortierella verticillata*
	***Basidiomycota***
**I.**	**Agaricales:***Agaricus bisporus, Amanita rubescens, Coprynopsis cinérea, Galerina marginata, Hypsizygus, marmoreus, Laccaria amethystina, L. bicolor, Lentinula edodes, Leucoagaricu sp, Moniliophthora roreri, Pleurotus ostreatus, Termitomyces sp, Schizophyllum commune*
	**Boletales:***Paxillus involutus, Serpula lacrymans*
	**Corticiales:***Punctularias trigosozonata*
	**Polyporales:***Dichomitus squalens, Fibroporia radiculosa, Gelatoporia subvermispora, Phanerochaete carnosa, Phlebiopsis gigantea, Postia placenta, Pycnoporus coccineus, Trametes cinnabarina, T. versicolor*
	**Russulales:***Heterobasidion irregular, Stereum hirsutum*
	**Gloeophyllales:***Gloeophyllum trabeum*
	**Jaapiales:***Jaapia argillacea*
	**Hymenochaetales:***Fomitiporia mediterránea, Schizopora paradoxa*
	**Sebacinales:***Serendipita indica*
	**Cantharellales:***Botryobasidium botryosum, Rhizoctonia solani*
	**Dacrymycetales:***Dacryopinax primogenitus*
	**Geastrales:***Sphaerobolus stellatus*
	**Auriculariales:***Auricularia subglabra*
	**Amylocorticiales:***Plicaturopsis crispa*
	**Tremellales:***Cryptococcus neoformans, C. gattii, Kockovaella imperatae, Naematelia encephala, Tremella mesenterica*
	**Trichosporonales:***Cutaneotrichosporon oleaginosum, Trichosporon asahii*
	**Cystofilobasidiales:***Xanthophyllomyces dendrorhous*
	**Wallemiales:***Wallemia mellicola*
**II.**	**Pucciniales:***Melampsora larici-populina, Puccinia sorghi, P. striiformis, P. graminis*
	**Mixiales:***Mixia osmundae*
	**Microbotryales:***Microbotryum intermedium, M. lychnidis-dioicae*
	**Leucosporidiales:***Leucosporidium creatinivorum*
	**Sporidiobolales:***Rhodotorula sp, R. graminis, R. toruloides, Sporidiobolus salmonicolor*
**III.**	**Ustilaginales:***Anthracocystis flocculosa, Kalmanozyma brasiliensis, Melanopsichium pennsylvanicum, Moesziomyces antarcticus, M. aphidis, Pseudozyma hubeiensis, Sporisorium reilianum, S. scitamineum, Ustilago hordei, U. maydis*
	**Ceraceosorales:***Ceraceosorus bombacis*
	**Malasseziales:***Malassezia pachydermatis, M. sympodialis*
	***Ascomycota***
**1.**	**Saccharomycetales:***Ascoidea rubescens, Brettanomyces bruxellensis, Candida albicans, C. auris, C. boidinii, C. dubliniensis, C. glabrata, C. maltosa, C. orthopsilosis, C. parapsilosis, C. tanzawaensis, C. tenuis, C. tropicalis, Clavispora lusitaniae, Cyber jadinii, C. mrakii, Debaryomyces fabryi, D. hansenii, Eremothecium cymbalariae, E. gossypii, Galactomyces candidum, Hanseniaspora uvarum, Hyphopichia burtonii, Kazachstania Africana, K. saulgeensis, Kluyveromyces lactis, K. marxianus, Komagataella phaffii, Lachancea lanzarotensis, L. quebecensis, L. thermotolerans, Lodderomyces elongisporus, Meyerozyma guilliermondii, Millerozyma farinosa, Naumovozyma castellii, N. dairenensis, Ogataea parapolymorpha, Pichia kudriavzevii, Saccharomyces arboricola, S. cerevisiae, Scheffersomyces stipitis, Spathaspora passalidarum, Sugiyamaellaligno habitans, Tetrapisispora blattae, T. phaffii, Torulaspora delbrueckii:Wickerhamomyces ciferrii, Yarrowia lipolytica, Zygosaccharomyces parabailii, Z. rouxii.*
**2.**	**Schizosaccharomycetales:***Schizosaccharomyces cryophilus, S. japonicus, S. octosporus, S. pombe*
**3.**	**Taphrinales:***Protomyces lactucaedebilis, Saitoella complicata*
	**Neolectales:***Neolecta irregularis*
	**Pneumocystidales:***Pneumocystis carinii, P. jirovecii: P, murina*
	**Pezizales:***Pyronema omphalodes*
	**Orbiliales:***Arthrobotrys oligospora, Dactylellina haptotyla, Drechslerella stenobrocha*
**4.**	**Hypocreales:***Acremonium chrysogenum, Beauveria bassiana, Claviceps purpurea, Cordyceps militaris, Drechmeria coniospora, Escovopsis weberi, Fusarium avenaceum, F. fujikuroi, F. graminearum, F. langsethiae, F. oxysporum, F. pseudograminearum, F, verticillioides, Hirsutella minnesotensis, Metarhizium acridum, M. anisopliae, M. guizhouense, M. robertsii, Nectria haematococca, Neonectria ditissima, Ophiocordyceps sinensis, Stachybotrys chartarum, Tolypocladium ophioglossoides, Torrubiella hemipterigena, Trichoderma atroviride, T, gamsii, T. guizhouense, T. harzianum, T. parareesei: T. reesei, T. virens, Ustilaginoidea virens.*
	**Microascales:***Ceratocystis platani, Scedosporium apiospermum, Thielaviopsis punctulata*
	**Glomerellales:***Colletotrichum chlorophyti, C. fioriniae, C. gloeosporioides, C. graminicola, C. higginsianum, C. nymphaeae, C. orbiculare, C. salicis, C. simmondsii, Verticillium dahliae*
	**Magnaporthales:***Gaeumannomyces tritici, Magnaporthe oryzae, Magnaporthiopsis poae*
	**Diaporthales:***Valsa mali*
	**Ophiostomatales:***Grosmannia clavigera, Ophiostoma piceae, Sporothrix brasiliensis, S. schenckii*
	**Sordariales:***Chaetomium globosum, C. thermophilum, Madurella mycetomatis, Neurospora crassa, N. tetrasperma, Thermothelomyces thermophila, Thielavia terrestris*
	**Xylariales:***Daldinia sp, Eutypa lata, Hypoxylon sp, Microdochium bolleyi, Pestalotiopsis fici, Pseudomassariella vexata, Rosellinia necatrix* Free, Adv Genet., 2013; 81:33–82
	**Togniniales:***Phaeoacremonium minimum*
**5.**	**Helotiales:***Botrytis cinerea, Glarea lozoyensis, Marssonina brunnea, M. coronariae, Phialocephala scopiformis, Sclerotinia borealis, S. sclerotiorum*
	**Erysiphales:***Blumeria graminis, Erysiphe necator*
**6.**	**Eurotiales:***Aspergillus aculeatus, A. clavatus, A. fischeri, A. flavus, A. fumigates, A. kawachii, A. lentulus, A. luchuensis, A. nidulans, A. niger, A. ochraceoroseus, A. parasiticus, A. rambellii, A. ruber, A. terreus, A. thermomutatus, A. turcosus, A. udagawae, Byssochlamys spectabilis, Elaphomyces granulates, Penicillium antarcticum, P.brasilianum, P.camemberti, P.coprophilum, P.decumbens, P.digitatum, P. expansum, P. flavigenum, P. freii, P. griseofulvum, P. italicum, P. nalgiovense, P. nordicum, P. oxalicum, P. roqueforti, P. rubens, P. solitum, P. steckii, P. vulpinum, Rasamsonia emersonii, Talaromyces atroroseus, T. islandicus, T. marneffei, T. stipitatus*
**7.**	**Onygenales:***Arthroderma otae, Blastomyces dermatitidis, Coccidioides immitis, C. posadasii, Emmonsia crescens, E. parva, Nannizzia gypsea, Paracoccidioides lutzii, P. brasiliensis:Trichophyton benhamiae, T. equinum, T. interdigitale, T. rubrum, T. soudanense, T. tonsurans, T. verrucosum*
**8.**	**Chaetothyriales:***Capronia coronata, C. epimyces, Cladophialophora bantiana, C. carrionii, C. immunda, C. psammophila, C. yegresii, Coniosporium apollinis, Cyphellophora europaea, Exophiala aquamarina, E. dermatitidis, E. mesophila, E. oligosperma, E. sideris, E. spinifera, E. xenobiotica, Fonsecaea multimorphosa, F. pedrosoi, Phialophora americana, P. attae, Rhinocladiella mackenziei*
	**Umbilicariales***: Umbilicaria pustulata*
	**Verrucariales:***Endocarpon pusillum*
**9.**	**Capnodiales:***Acidomyces richmondensis, Baudoinia panamericana, Dothistroma septosporum, Hortaea werneckii, Mycosphaerella eumusae, Pseudocercospora fijiensis, P. musae, Rachicladosporium antarcticum, Sphaerulina musiva, Zymoseptoria brevis, Z, tritici*
	**Dothideales,***Aureobasidium melanogenum, A. namibiae, A. pullulans, A. subglaciale*
	**Venturiales:***Verruconis gallopava*
	**Botryosphaeriales:***Diplodia corticola, Macrophomina phaseolina, Neofusicoccum parvum*
	*Phaeomoniellales: Phaeomoniella chlamydospora*
	**Pleosporales***: Alternaria alternata, A. infectoria, Ascochyta rabiei, Bipolaris zeicola, B. victoriae, B. oryzae, B. sorokiniana, B. maydis, Clohesyomyces aquaticus, Coniosporium apollinis, Epicoccum nigrum, Leptosphaeria maculans, Parastagonospora nodorum, Pyrenophora teres, P. tritici-repentis, Setosphaeria turcica, Stemphylium lycopersici*

### 4.6 Synthesis of β 1,6 glucans*.*

Previously we mentioned that most of the fungal β 1,3 glucans are branched polysaccharides joined mainly through β 1,6 linkages (see above). As occurs with other glucans, most studies of these polysaccharides and their synthesis have been concentrated on *S. cerevisiae*. By use of a clever method, the putative genes encoding proteins involved in the synthesis of β 1,6 glucans were identified (reviewed by Shahinian and Bussey, 2000). The method involved the isolation of mutants resistant to the *S. cerevisiae* K1 killer protein, on the premises that this binds to β 1,6 glucans, to further on kill the sensitive yeasts. Interestingly, Santos, et al., (2000) demonstrated that the killer toxin produced by the strain of *Pichia membranifaciens* CYC1106, also binds to β 1,6 glucans.

Analyzing the complementation groups of the *S. cerevisiae* mutants resistant to the yeast K1 killer toxin, ten genes were identified, and the cellular location of the codified products was determined (Brown et al., 1993, Brown and Gordon, 2003); for a review see Shahinian and Bussey (2000). These are the following: *KRE1*, a gene encoding a soluble cytoplasmic protein; genes encoding endoplasmic reticulum (ER) soluble proteins *KRE5* and *ROT2/GLS2*, and *CNE1* encoding a a membrane bound protein; genes encoding membrane Golgi proteins: *KRE6* and *SKN1*; a gene encoding a plasma membrane GPI protein: *KNH1*, and genes encoding periplasmic soluble proteins, *KRE9* and *KNH1*. Despite these important results, the specific roles of the proteins encoded by these genes remains mostly hypothetical, and apparently none of them corresponds to the indispensable β 1,6 glycosyl transferase. In addition, it must be indicated that *in silico* analysis of Basidiomycota species identified homologs only of *KRE1, KRE9, KRE11*, and *CNE1*, whereas in Zygomygota and Chitridimycota species, these were reduced to *KRE6* and *SKN1* (Ruiz-Herrera and Ortiz-Castellanos, 2010). In this sense, Gilbert et al. (2010) characterized seven *KRE* genes from *C. neoformans*, and demonstrated that only a *KRE5* deletion mutant and a double mutant in *KRE6,* and *SKN1* were deficient in the levels of β 1,6 glucans, and showed alterations in growth, morphology, cell wall structure, and virulence. Pan et al. (2017) described that spores from *S. cerevisiae* mutants defective in *KRE1*, *KRE9* and *BIG1*, but not in *KRE6* were sensitive to ether, an indication that their structures were affected. All these results are interesting, since they almost coincide with the only homolog genes identified in Zygomycota and Chitridiomycota.

Taking into consideration that these genes encode proteins located at the ER and Golgi complex, it would appear that the synthesis of β 1,6 glucans is initiated intracellularly, and that the precursors are transported to the surface to bind to the β 1,3 glucan chains. Alternatively, the first step in β 1,3, β 1,6 glucans synthesis may involve the synthesis of the hypothetical protein acceptor, suggested above, at the ER. This process would be followed by the synthesis of the polysaccharide chains and branching at later steps of the secretory pathway and at the cell surface.

## 5 The possible role of members of the glucosylhydrolase family 72 on glucan synthesis*.*

A family of fungal GPI (glycosylphosphatidylinositol) proteins with glucosyl transferase activity has been described to be active in some transfer reactions that affect the structure of the cell wall beta glucans. These enzymes have been classified into the glucosylhydrolase family 72 (see reviews by Mouyna et al., 2000, Popolo et al., 2001). In general, these proteins have the capacity to (in a similar way to the glycogen branching enzymes) break a β 1,3 glucan molecule, and transfer the residue obtained to the non-reducing end of different β 1,3 glucan chains whose size increases accordingly. The exceptions to this rule are, *i*) an enzyme from *C. albicans* that had the capacity to split a laminaribiose fragment from the reducing end of a β 1,3 glucan chain, and transfer it to another β 1,3 glucan molecule to form the branch of a β 1,6-linked molecule (Hartland et al., 1991), *ii*) an enzyme with a similar mode of action described in *A. fumigatus* by Gastebois et al. (2009). Unfortunately whether these enzymes were the long-searched β 1,6 branching enzyme has not been further confirmed or denied. The other exception, (*iii*), was an enzyme from *S. cerevisiae* and *C. albicans* that was able to transfer a fragment from a β 1,3 glucan to the non-reducing end of another β 1,3 glucan chain to form a β 1,6 bond.

The physiological role of these anzymes, regarding the synthesis of β 1,3, and/or β 1,6 glucans, is uncertain. Taking into consideration that they are GPI-proteins, and accordingly are located at the plasmalemma with their orientation towards the cell wall, it has been speculated that they may be involved, not on the synthesis, but on the linking and modifications of the wall glucans. However, more recently different results were obtained by Almanianda et al. (2017). These authors described data showing that Bgl2p and mainly Gas1p were involved in β 1,6 branching of linear β 1,3 glucan oligomers, followed by β 1,3 elongation of the branches.

## 6 Concluding remarks

As it is discussed in the present chapter review, glucans are the carbohydrates more abundant in the living beings, and fungi are not the exception. In fungi, glucans fulfill the roles of: reserve of energy, structural, recognition or signaling, and protection. The two groups of glucans present in fungi are made of glucose bound through alpha or beta linkages, mainly either α 1,3, and α 1,6, or β 1,3 and β 1,6. Both kinds of glucans are the most abundant polysaccharide in the fungal cell wall, where they act as structural elements. Also in common, the substrate for the corresponding synthases is UDPGlc. Interestingly α 1,3, glucans are present only in the Dikarya clade, suggesting that their synthesizing enzymes probably aroused during evolution from the modification of the genes encoding another glycosyl transferase.

β 1,3, glucan synthases are present in all fungal phyla witht the exception of Microsporidia, and have similarities to the enzymes that synthesize β 1,3, glucans in plants, algae and Chromista, suggesting that the fungal genes were possibly horizontally transferred from a member of the Plant or Chromista kingdoms. The variability in the number of glucan synthesizing genes present in the several fungal species is an indication that duplications and losses occurred during evolution. It is also noticeable, that in contrast to α 1,3 and β 1,3, glucans, the mechanism of synthesis of β 1,6 glucans remains rather unknown, although a number of genes possibly somehow related to the process have been identified, but not their precise roles.

In summary, it is evident that although a number of important aspects on glucan synthesis in fungi are still unknown or unexplored, our knowledge on the distribution, structure, mechanism of synthesis, roles, and evolution of the different glucans in fungi has been widely expanded in recent years.

## Declaration of Competing Interest

The authors declare that they have no known competing financial interests or personal relationships that could have appeared to influence the work reported in this paper.
